# In vitro evaluation of the susceptibility of *Acinetobacter baumannii* isolates to antiseptics and disinfectants: comparison between clinical and environmental isolates

**DOI:** 10.1186/s13756-017-0195-y

**Published:** 2017-04-11

**Authors:** Sanae Lanjri, Jean Uwingabiye, Mohammed Frikh, Lina Abdellatifi, Jalal Kasouati, Adil Maleb, Abdelouahed Bait, Abdelhay Lemnouer, Mostafa Elouennass

**Affiliations:** Department of Clinical Bacteriology, Mohammed V Military teaching hospital, research team of Epidemiology and Bacterial resistance, Faculty of Medicine and Pharmacy, Mohammed V University, Rabat, Morocco

**Keywords:** *Acinetobacter baumannii*, Resistance, Antiseptics, Disinfectants

## Abstract

**Background:**

This study aims to assess the susceptibility of *Acinetobacter baumannii* isolates to the antiseptics and disinfectants commonly used, and to the non-approved product.

**Methods:**

This is a prospective study carried out from February to August 2015, in the Bacteriology department of Mohammed V Military Teaching hospital of Rabat on *A.baumannii* isolates collected from colonized and/or infected patients and environmental samples. The antiseptics and disinfectants susceptibility testing was assessed using the micromethod validated in our department. The antiseptics and disinfectants studied were: 70% ethyl alcohol, chlorhexidine, povidone-iodine, didecyldimethylammonium chloride and a commercial product which was presented as a hospital disinfectant (non-registered product).

**Results:**

Povidone-iodine, 0.5% chlorhexidine digluconate, 70% ethyl alcohol and didecyl dimethyl ammonium chloride in combination with N- (3-aminopropyl) -N-dodecylpropane-1, 3-diamine were effective against all the 81 *A.baumannii* isolates tested, and their logarithmic reduction ≥ 5 were observed in 100% of the isolates in their undiluted form. The strains isolated from patients were more resistant than environmental strains: at a dilution of ½ for 70% ethyl alcohol (37.77% vs 11.11%, *p* = 0.007) and at a dilution of 1/10 (100% vs 69.44%, *p* < 0.001) for povidone iodine. The non-registered product was ineffective with a resistance rate of 96.29% at a dilution of 1/50, 45.67% at a dilution of 1/10 and 13.58% in its purest form.

**Conclusion:**

Our study revealed the effectiveness of the main disinfectants and antiseptics used in Morocco; three antiseptics tested were effective in their purest form against the 81 *A.baumannii* isolates. Regarding disinfectants, our results showed an efficacy of didecyl dimethyl ammonium at the recommended use concentration and in its purest form. This study emphasizes the need for using disinfectants and antiseptics in dilutions recommended by the manufacturer because the insufficient dilutions of these products are not effective. Our findings also demonstrated an inefficiency of the non-registered product against *A.baumanii* isolates. However, the non-registered products should be prohibited.

## Background

*Acinetobacter baumannii* is a Gram-negative bacillus that has emerged in recent years as one of the pathogens that poses the most problems of antibiotic resistance, morbidity and mortality in health facilities worldwide [1].

*A.baumannii* is a ubiquitous bacterium that can be isolated from both the environment [2] and the human’s skin [3, 4]. It mainly affects debilitated patients [5] and it is responsible for a variety of life-threatening infections including bacteremia and pneumonia [1]. In an American study, 5 to 10% of nosocomial pneumonia and 1.3% of bacteremia were due to *A. baumannii* [6, 7]. In a Moroccan study published in 2016, *Acinetobacter spp*. isolates represented 6.94% of all bacterial clinical isolates and 9.6% of Gram negative rods [8].

The remarkable ability of *A.baumannii* to easily acquire resistance factors ranked it among the organisms that threaten the current therapeutic armamentarium. *A. baumannii* strain resistant to all known antibiotics has already been reported in France [9]. The Moroccan studies showed that the resistance rate to imipenem, ceftazidime, amikacin and ciprofloxacin increased from 23 to 76%, 63 to 86%, 41 to 52% and 68 to 87% respectively during the last 13 years [8, 10].

The importance of hand transmission in the spread of this bacteria and prolonged survival in the environment (>8 days) complicates the prevention and control of these infections [1]. Indeed, hand hygiene and environmental cleanliness are the keystone of the infection control measures for the prevention of the spread of *A.bauamannii*. The use of antiseptics and disinfectants is an important means in these infection control strategies. Their use allows the reduction or elimination of bacterial reservoir and prevents the transition from colonization to infection [11].

Contrary to antibiotics, studies that have been devoted to the emergence of *A. baumannii* resistant to antiseptics and disinfectants are very few. Some emphasize the increase in resistance rates to these products. A study assessing the susceptibility of 445 bacterial strains from various genera against more than 50 types of disinfectants revealed that *A. baumannii* isolates were the most resistant species. Resistance rates were higher against oxidizing agents while the lowest resistance rate was observed against quaternary ammonium compounds and amines [12].

Antiseptic resistance rates vary. One study showed that on hands artificially contaminated with *A. baumannii,* ethyl alcohol and povidone-iodine had higher removal ratio than chlorhexidine and ordinary soap [13].

The aim of our study was to assess the susceptibility of *A. baumannii* strains to the antiseptics and disinfectants commonly used, and to a non-registered product.

## Methods

### Type of the study

This is a prospective study performed in the Bacteriology department of Mohammed V Military Teaching hospital (HMIMV) of Rabat from February to August 2015. HMIMV has a hospital capacity of more than 700 beds and contains 2 intensive care units (medical and surgical) with 10 beds each, a center for burns treatment, surgical and medical units, and laboratory and imagery departments.

### Bacterial isolates

The *A. baumannii* strains tested were isolated from colonized and/or infected patients, and environmental samples in the HMIMV.

In addition to these isolates, a reference strain was tested (*Escherichia coli ATCC® 25922™*).

### Antiseptics and disinfectants tested

In our institution, the antiseptics mainly used are polividone iodine and chlorhexidine. For the bio-cleaning of floors and surfaces, the disinfectant used is didecyl dimethyl ammonium chloride.

During our study, five products were tested including three antiseptics and two disinfectants including a commercial detergent-disinfectant whose composition is unknown (Table 1).

**Table 1:** The recommended dilutions, abbreviation, active ingredient and the dilutions tested for each product in our study

Product	Abbreviation	Active ingredient	Recommended dilutions	dilutions tested
*Betadine scrub®*	PVPI	4% povidone-iodine	Pure, 1|3	pure, 1|3, 1|10, 1|100, 1|1000
*septeal®*	CHX	0.5% chlorhexidine digluconate	Pure	pure, 1|10, 1|100, 1|1000
ethyl alcohol 70	AL	70% ethyl Alcohol	Pure	pure, 1|2, 1|10, 1|100, 1|1000
*Surfanios®*	DDA	N- (3-aminopropyl) -N-dodécylpropane-1, 3-diamine (51 mg/g) + didecyl dimethyl ammonium chloride (25 mg/g)	1|400 (0,25%)	pure, 1|10, 1|100, 1|200, 1|400, 1|1000, 1|10000, 1|1000000
Non registered product	NRP	unknown composition	1|50 (2%)	pure, 1|10, 1|50, 1|100, 1|1000

### Methods

The sensitivity of *A.baumannii* isolates to antiseptics and disinfectants was assessed using the microdilution method (micromethod), validated in our laboratory.

The validation of our method was made by comparing its results with those obtained by the tube dilution method (macromethod) for three isolates to Surfanios®, as it was described by Mama et al. (2014) [14].

### Validation of a micromethod for the determination of the susceptibility to antiseptics and disinfectants

#### Inoculum preparation

After thawing the bacterial isolates to be tested, a brain heart broth was inoculated and incubated at 37 °C for 18 to 24 h. The broths were subsequently subcultured on blood agar and incubated at 37 °C for 18 to 24 h. The inoculum was prepared by saline suspension of isolated colonies selected from an 18 to 24 h agar plate. The turbidity of these suspensions was adjusted to. 0.5 McFarland standard.

#### Dilution of the initial inoculum

After calculating the different volumes to be used for the micromethod by respecting the proportions used in the macromethod, the volume of the bacterial suspension was found very low (1 μl). To increase the volume, 1/10 dilution of the previously standardized bacterial suspension at 0.5 McFarland was performed.

### Preparation of antiseptic and disinfectant solutions

The different prepared dilutions of each product were chosen according to the concentration recommended by the manufacturer (Table 1).

### Validation tests

#### Macromethod validation

In a sterile test tube, 4.9 ml of sterile trypticase soy broth was mixed with 5 ml of each dilution of the antimicrobial agent. To obtain a final volume of 10 ml, 0.1 ml of standardized bacterial suspension was inoculated into each tube.

Two control tubes were used; positive control tube containing only the nutrient broth and the strain to be tested, while negative controls were established as follows: nutrient broth only, nutrient broth and antimicrobial agent.

After 24 h at 37 °C, the visible signs of bacterial growth (turbidity) were not possible to detect given the macroscopic qualities of the product-broth mixture in all the tubes including the control tubes. All tubes were then subcultured on Bromocresol Purple (BCP) agar and incubated at 37 °C for 24 h. The reading of each culture plate has been validated by the absence of growth in sterility control wells and bacterial growth in fertility control wells.

#### Micromethod validation, and antiseptics and disinfectants susceptibility testing

The wells of a microplate were filled with appropriate volumes of increasing concentrations of antiseptic or disinfectant solution and then completed with corresponding volumes of broth and suspension of the inoculum.

In practice, each well of a sterile 96-well plate was inoculated with 50 μl of dilution of the antimicrobial agent, 40 μl of trypticase soy and 10 μl of diluted bacterial suspension.

The positive control wells were inoculated with 40 μl of nutrient broth and 10 μl of the bacterial suspension (fertility testing), while the negative controls were inoculated with nutrient broth and the antimicrobial agent (sterility testing).

After 24 h at 37 °C, all tubes were then subcultured on BCP agar and incubated at 37 °C for 24 h. Reading of agar plates was performed as described in macromethod validation.

The macromethod was used as reference for validating the micromethod, with both methods leading to the same results.

The evaluation of the *A. baumanni* susceptilibity to antiseptics and disinfectants was realized by using micromethod as it was described in the validation’s paragraph. The number of colony-forming units was counted on each plate. The plates on which manual counting of bacterial colony-forming units was not possible, the number of colony-forming unit was considered to be above 10^7^.

The rate of the log reduction of the different products was calculated. The ⩾5 log10 reduction was considered as significant reduction of growth.

### Statistical analysis

Statistical analysis was performed using SPSS statistical software version 13.0 and Microsoft Office Excel 2007. Susceptibility comparisons of the isolates from the patient and environmental samples to different products were established using the chi test-square. *P* values less than 0.05 were considered statistically significant.

## Results

During this study, 81 *A.baumannii* isolates were tested: 45 strains from patient samples and 36 strains from the environmental samples. The majority of clinical isolates were predominantly collected from intensive care unit’s (ICUs) patients (*n* = 34, 75.5%), followed by those from medical department (*n* = 8, 17.8%) and external consultants (*n* = 3, 6.7%). Of the clinical isolates, 23 (51.1%) were colonizing isolates and 22 (48.9%) were infectious ones. The colonizing isolates were isolated from anal margin (43.5%), groin area (8.34.8%) and mouth (n21.7%), while the isolation sites of infectious isolates were: broncho-pulmonary (45.5%), urine (31.8%) and blood culture (13.6%), catheters (4.5%), joint fluid (4.5%) and cutaneous lesions (4.5%). The environmental isolates were exclusively recovered from the ICUs. The distribution of isolation sites for environmental isolates was: bed sheets (38.9%), soil (36.1%), hospital respirators (11.1%), trolleys (5.6%), gallows (2.8%), pillows (2.8%) and monitors (2.8%).

All chemical agents tested were found to be effective on the *Escherichia coli ATCC 25922* reference strain in their purest form (Table 2).

**Table 2:** Efficacy of antiseptics and disinfectants tested on *Escherichia coli ATCC® 25922™* reference strain

Product	Dilution	Bacterial growth
PVPI	PURE	-
	1|3	-
	1|10	+
	1|100, 1|1000	+
CHX	PURE	-
	1|10	-
	1|100	-
	1|1000	+
AL	PURE	-
	1|2	+
	1|10	+
	1|100, 1|1000	+
DDA	PURE, 1|10, 1|100, 1|400	-
	1|1000	-
	10–4	+
	10–5, 10–6	+
NRP	PURE	-
	1|10	+
	1|50	+
	1|100, 1|1000	+

*PVPI* povidone-iodine, *CHX* chlorhexidine digluconate, *AL* ethyl Alcohol, *DDA* N-(3-aminopropyl)-N-dodécylpropane-1,3-diamine + didecyl dimethyl ammonium chloride, *NRP* non registered product, *(+)* Growth, *(-)* No growth

The Tables 3 and 4 represent respectively the resistance rates of *A.baumannii* strains and the logarithmic reduction ≥ 5 over the initial time zero inoculum levels of the various products tested. Povidone-iodine, 0.5% chlorhexidine digluconate, 70% ethyl alcohol and didecyl dimethyl ammonium chloride in combination with N- (3-aminopropyl) -N-dodecylpropane-1, 3-diamine were effective against all the 81 *A.baumannii* isolates tested and their logarithmic reduction ≥ 5 was observed in 100% of isolates in their undiluted form. The strains isolated from patients were more resistant than environmental strains: at a dilution of ½ for70 % ethyl alcohol (37.77% vs 11.11% and *p* = 0.007) and at a dilution of 1/10 (100% vs 69.44%, *p* < 0.001) for povidone iodine. The non-registered product was ineffective with resistant rate of 96.29% at a dilution of 1/50, 45.67% at a dilution of 1/10 and 13.58% in its purest form.

**Table 3:** Comparison of resistance rates between *A.baumannii* strains isolated from patients and from environmental samples to different products tested

Products	Dilution	Origin of the strains		Total (*n* = 81)	*P*
		Patients (*n* = 45)	Environment (*n* = 36)		
PVPI	PURE	0% (0)	0% (0)	0% (0)	-
	1|3	24.44% (11)	11.11% (4)	18.51% (15)	0.125
	1|10	100% (45)	69.44% (25)	86.41%(70)	<0.001
	1|100, 1|1000	100% (45)	100% (36)	100% (81)	-
CHX	PURE	0% (0)	0% (0)	0% (0)	-
	1|10	13.33% (6)	0% (0)	7.40% (6)	0.31
	1|100	51.11% (23)	66.66% (24)	58.02 (47)	0.159
	1|1000	100% (45)	100% (36)	100% (81)	-
AL	PURE	0% (0)	0% (0)	0% (0)	-
	1|2	37.77% (17)	11.11% (4)	25.92% (21)	0.007
	1|10	93.33% (42)	80.55% (29)	87.65% (71)	0.100
	1|100, 1|1000	100%(45)	100% (36)	100% (81)	-
DDA	PURE, 1|10,1|100, 1|400	0% (0)	0% (0)	0% (0)	-
	1|1000	24.44% (11)	8.33% (3)	17.28% (14)	0.057
	10–4	86.66% (39)	69.44% (25)	79.01% (64)	0.059
	10–5, 10–6	100% (45)	100% (36)	100%(81)	-
NRP	PURE	24.44% (11)	0% (0)	13.58% (11)	0.001
	1|10	66.66% (30)	19.44% (7)	45.67% (37)	<0.001
	1|50	97.77% (44)	94.44%(34)	96.29% (78)	0.582
	1|100, 1|1000	100% (45)	100% (36)	100% (81)	-

*PVPI* povidone-iodine, *CHX* chlorhexidine digluconate, *AL* ethyl Alcohol, *DDA* N-(3-aminopropyl)-N-dodécylpropane-1,3-diamine + didecyl dimethyl ammonium chloride, *NRP* non registered

- : *p* value not calculated

**Table 4:** The rate of isolates presenting logarithmic reduction ≥ 5 from initial inoculum

Product	Dilution	The rate of isolates presenting logarithmic ≥ 5 Log_10_ reduction
PVPI	PURE	100%
	1|3	81.48%
	1|10	13.58%
	1|100, 1|1000	0%
CHX	PURE	100%
	1|10	92.59%
	1|100	41.97%
	1|1000	0%
AL	PURE	100%
	1|2	74.07%
	1|10	12.34%
	1|100, 1|1000	0%
DDA	PURE, 1|10, 1|100, 1|400	100%
	1|1000	82.71%
	10–4	20.98%
	10–5, 10–6	0%
NRP	PURE	86.4%
	1|10	54.32%
	1|50	3.70%
	1|100, 1|1000	0%

*PVPI* povidone-iodine, *CHX* chlorhexidine digluconate, *AL* ethyl Alcohol, *DDA* N-(3-aminopropyl)-N-dodécylpropane-1,3-diamine + didecyl dimethyl ammonium chloride, *NRP* non registered product

## Discussion

Our study was focused on three commonly used antiseptics: 70% ethyl alcohol, chlorhexidine and povidone-iodine, on a disinfectant (didecyldimethylammonium chloride) and on a commercial product which was presented as a hospital disinfectant.

The 70% ethyl alcohol is used for antisepsis of small superficial wounds and skin before an injection. It’s a component of several hand sanitizers. Other alcohols, such as propanol and isopropanol, are also used as hand sanitizers [11]. In our study, pure ethyl alcohol (70%) was effective against all *A.baumannii* isolates. At a dilution of 1/2, the resistance rate was 25.92%. The strains isolated from patients were more resistant than environmental strains (37.77% vs 11, 11%, *p* = 0.007). The ⩾5 log10 reduction was obtained in 100% of isolates at undiluted state, in 74% at dilution of ½ and in 12.34% at dilution of 1/10. Wisplinghoff et al. [15] used 60% propanol and found the results similar to ours while the contact time was 15 s, 30s, 60s and 120 s.

The 0.5% chlorhexidine digluconate is recommended in antisepsis of surgical wounds, common skin infections, and antisepsis of healthy skin prior to minor surgery. It is applied to the skin without rinsing [11]. In our study, chlorhexidine at its undiluted state has shown its effectiveness against all 81 isolates tested. The logarithmic reduction ≥ 5 was observed in 100% of isolates in its undiluted state and in 92.59% of isolates at a dilution of 1/10. The difference in resistance rates between isolates from the patient and the environmental samples was not significant. Comparing these rates with those of 70% ethyl alcohol, the chlorehexidine is more effective. Our results agree with Wisplinghoff et al’s study [15]. Another study showed that daily bathing with chlorhexidine 2% in the ICU reduced the rate of acquisition of *A.baumannii* isolates resistant to carbapenems by 51.8% [16].

The povidone iodine is a foam solution composed of iodine and is used for scrubbing, antisepsis of healthy skin, preoperative shower; for which, it should be used in its purest form. For the cleansing of contaminated wounds, it is recommended that one-third be diluted with water [11]. In our study, all isolates were sensitive to pure povidone-iodine. While at the dilution of 1/3(dilution recommended for cleansing of wounds), 18.51% isolates were resistant. In Povidone Iodine’s purest form and at a dilution of 1/3, the difference in resistance rates between isolates from patients and the environmental isolates was not significant. Clinical strains were more resistant than the environmental strains at a dilution of 1/10(100% vs 69.44%, *p* < 0.05). The logarithmic reduction ≥ 5 was observed in 100% in its purest form, in 81.48% at the dilution of 1/3 and in 13.58% at a dilution of 1/10. In the study conducted by Wisplinghoff et al [15], pure povidone iodine was active against all *Acinetobacter* isolates tested.

Environmental hygiene plays an important role in cross-transmission of *A. baumannii*. Prolonged survival of *A.baumannii* in a dry environment has been correlated with ongoing epidemics in ICUs [17]. During an *A. baumannii* outbreak which occurred over a period of 14 months in a neurosurgery ICU in the United Kingdom, a significant correlation was observed between the number of infected or colonized patients and the number of environmental isolates (*p* = 0.004) [18]. The majority of detergent-disinfectants for floors and surfaces are composed of quaternary ammonium compounds only [19] or associated with other substances (Biguanide derivatives, aldehydes, isopropanol, alkylamine, amino acid, hydrogen peroxide, ethanol …). Some studies reported acquired resistance to quaternary ammonium compounds, and that these products have a higher effectiveness against Gram-positive bacteria than against Gram-negative bacteria [11, 20]. Didecyldimethylammonium chloride in association with the amino acid hydrochloride (Surfanios®) is used in the hospital, diluted to 0.25%(1/400) according to the manufacturer’s recommendations, for the biocleaning of floors and surfaces. In our study, Surfanios® was effective against all isolates at the recommended dilution (1/400). Resistance was observed at a dilution of 1/1000 with a rate of 17.28%. There was no significant difference in resistance between the clinical and environmental isolates. In the study of Reichel et al [21], three Quaternary ammonium compound products were effective against three *A. baumannii* strains.

Another product tested in this study, is presented as a hospital-level disinfectant and its composition is unknown (non-registered product). The concentration recommended by the manufacturer is 2% (1/50). This product was ineffective with resistance rate of 96.29% at a dilution of 1/50, 45.67% at a dilution of 1/10 and 13.58% in its purest form. Our results show the ineffectiveness of this product and emphasize the advantage of using the products inscribed on the positive lists published by learned societies. The right choice of antiseptics and disinfectants plays a crucial role in preventing the development of resistance.

Globally, the clinical isolates were more resistant to the antiseptics and disinfectants in their diluted state than the environmental ones. This shows that the minimum inhibitory concentrations of clinical and those of environmental isolates to biocides are not the same. The colonizing and infecting *A.baumannii* isolates were the most resistant to the antiseptics and disinfectants. This can probably be explained by the selection pressure exerted by the use of antiseptics and sometimes facilitated by the sub-inhibitory concentrations [22].

## Conclusion

Our study revealed the effectiveness of the main disinfectants and antiseptics used in Morocco: three antiseptics tested were effective in their purest form against the 81 *A.baumannii* isolates. However, with povidone iodine diluted at one third, 18.51% of isolates were resistant. Regarding disinfectants, our results showed an efficacy of Didecyldimethylammonium at the recommended concentration. This study emphasizes the need for using disinfectants and antiseptics in dilutions recommended by the manufacturer because the insufficient concentrations of these products are ineffective. Our findings also demonstrated an inefficiency of the non-registered product against Acinetobacter isolates. The market for these products is constantly increasing but it is potentially associated with risks. Therefore, a regulatory framework for the use of these products must be implemented both for hospital and community use.

## Abbreviations

*A.baumannii*: *Acinetobacter baumannii*
AL: Ethyl alcohol
BCP: Bromocresol purple
CHX: Chlorhexidine digluconate
DDA: N-(3-aminopropyl)-N-dodécylpropane-1,3-diamine + didecyl dimethyl ammonium chloride
HMIMV: *Mohammed V military teaching hospital of Rabat*
ICUs: Intensive care units
NRP: Non registered
PVPI: Povidone-iodine
